# High level of RNF187 contributes to the progression and drug resistance of osteosarcoma

**DOI:** 10.7150/jca.33488

**Published:** 2020-01-01

**Authors:** Wen-Bing Wan, Kai Wu, Kun Peng, Zhi-Qiang Qiu, Zhi-Bin Duan, Xiang Chen, Ze-Min Xu, Ke Cheng, Jiang-Ming-Hao Zhao, Qing-Ming Shi

**Affiliations:** Department of Orthopedic Surgery, The Second Affiliated Hospital of Nanchang University, Nanchang, Jiangxi 330006, PR China.

**Keywords:** Osteosarcoma, RNF187, Drugs resistance, Prognosis

## Abstract

**Objectives:** Ring finger protein 187 (RNF187) was recently demonstrated to be up-regulation and function as a promoter in multiple cancers. However, the roles of RNF187 in osteosarcoma (OS) are unclear. Here, we tried to reveal the clinicopathological and biological roles of RNF187 in OS.

**Materials and Methods:** We employed the quantitative real-time polymerase chain reaction (qRT-PCR) and immunohistochemistry (IHC) to determine the expression of RNF187 in OS tissues and cells. Migration and invasion capacities were analyzed by wound healing and transwell assays, and colony formation and CCK8 assays were performed to investigate proliferative ability. The functional effects of RNF187 on OS drugs resistance were further determined by CCK8 and western blot assays. Then, the relationship between RNF187 expression and clinical implications was analyzed by tissue microarrays (TMAs) including 51 OS cases. Moreover, the prognostic value was also determined by Kaplan-Meier analysis.

**Results:** We reported that RNF187 mRNA was significantly increased in OS tissues compared to matched nontumorous tissues (3.83 ±0.79 vs. 1.70 ± 0.63), which was in line with the IHC assay in TMAs. By RNA interference and cDNA transfection, we showed high level of RNF187 increased the migration, invasion and proliferation of OS cells. Moreover, we demonstrated that elevated RNF187 expression induced OS cell drugs resistance, activated the ERK1/2 molecular and markedly enhanced the BCL-2 expression. Clinically, OS patients with high level of RNF187 was associated with Histologic differentiation (p=0.001), an advanced Enneking stage (p=0.001), response to chemotherapy (p=0.004), and metastasis (p= 0.001). Clinically, our data displayed that the RNF187 overexpression in OS samples associated with shorten overall survival (p=0.001) and high tumor recurrence (p=0.001) in postoperative OS patients.

**Conclusions:** Our results indicate that Elevated RNF187 expression is a new adverse outcomes marker for OS patients and may be used as a new therapeutic target of OS.

## Introduction

Osteosarcoma (OS) is the most common type of primary malignant neoplasm of the skeleton in children and young adolescents 1. Despite significant advances have been achieved in chemotherapeutics and surgical management, the long-term survival for OS patients remains dismally poor. Now near 30-40 percent of patients after the successful resection of primary tumors may develop metastases, moreover approximately 80 percent of the OS patients with metastatic disease at diagnosis 2. Generally, the standard multimodal therapy failure for OS is associated with a very poor prognosis. Thus, it is imperative to reveal novel molecular markers in the diagnosis, treatment and prognosis of osteosarcoma 3.

Ubiquitination is involved in post-translational modification and plays vital roles in different biological and pathological processes in eukaryotes 4. E3 ubiquitin ligases (E3s) are critical importance due to not only transfer ubiquitin to specific substrates 5. Ring finger protein 187 (RNF187), a RING domain-containing ubiquitin E3 ligase, was lately verified to overexpressed in hepatocellular carcinoma and non-small cell lung carcinoma and advanced tumor development by inducing tumor cell epithelial-mesenchymal transition (EMT) 6, 7. Furthermore, RNF187 knockout was revealed to suppress the cellular proliferation via inhibiting the transcription of AP-1-related genes, and RNF187 overexpression activated Wnt and Ras pathways to accelerate the tumor formation in colon epithelial tumor 8. Moreover, RNF187 was originally demonstrated to be a co-activator of JunD by a yeast two-hybrid screen 9. Together, the aforementioned data indicate that the abnormal RNF187 may be an important booster in tumor development 10. However, the expression and roles of RNF187 in OS are largely uncharacterized.

Here, we tried to determine the RNF187 expression in OS tissues and cells, and assess the role of RNF187 in biological behaviors of OS cells via RNF187 knockdown and cDNA transfection. Finally, the clinical implications of RNF187 in OS patients were studied in TMAs including 51 OS cases.

## Materials and Methods

### Cell lines, plasmids and chemotherapeutic agents

OS cells lines , U-2OS, MG-63, Saos and HOS, and a human osteoblastic cell line, hFOB1.19 (from the Cell bank of Chinese Academy, Shanghai, China), were conventionally cultured in DMEM and RPMI 1640 (Gibco; Waltham, MA, USA) supplied with 10 percent heat-inactivated fetal calf serum (FCS) (Gibco; Waltham, MA, USA), 1% L-glutamine, and penicillin/streptomycin (10,000 U/mL and 10,000 µg/mL) (Yesen, Shanghai, China), and kept at 37°C in a humidified incubator under 5% CO2.

The pGPU6-GFP-vshRNA-RNF187s and pGMLV-RFP-cDNA-RNF187 was constructed by genomeditech biological company (Genomeditech, Shanghai, China). Paclitaxel (Yesen, Shanghai, China) and Docetaxel (Yesen, Shanghai, China) were kept in our laboratory.

### Patients and follow-up

The data of OS patients who underwent surgical therapy at the Second Affiliated Hospital of Nanchang University between January 2006 and December 2012 were summarized and re-examined according to our previous study 11. The pathological diagnosis was affirmed by two pathologists. The frozen specimens were storage at -80°C. Paraffin-embedded OS samples were conventionally prepared by the pathologist and kept in the department of pathology of our hospital. Written agreement from patients or their guardians was obtained and approved by the Nanchang University Ethics Committee. Clinicopathologic features of the present series of OS patients are shown in** Table 1**.

### RNA Extraction and *q*RT-PCR

Fifteen OS and their matched nontumorous specimens were used to determine the RNF187 expression, and the RNAs were extracted routinely according to the description elsewhere 12. The DNA amplification and revelation were accomplished by applying the ABI PRISM 7900 Sequence Detection System (Applied Biosystems, Foster City, CA). β-action was availed as an internal reference. Primers for RNF187 were: 5'-TGGAAATCATGAGAACTTG-3' and: 5'-ACGGTCCATCACGTGTCC-3′; and β-action: 5'-AGAGCTACGAGCTGCCTGAC-3' and 5'-AGCACTGTGTTGGCGTACAG-3'. The PCR amplification were in accordance with our previous study 11. This test was repeated three times.

### Western blot

Thirty mg protein extracted from the OS cell lines were used for western blot as described elsewhere 13, 14. All antibodies used in this study were listed in **supplementary Table 1**. β-action (1:2000, Abcam, Cambridge, UK) was used as an internal reference. This test was repeated in triplicate. All the antibodies employed in our experiment were displayed in**supplementary Table 1**.

### Cell proliferation, cell migration, and invasion assays

Cell proliferation was determined by the Cell Counting Kit-8 (CCK8) (Yesen, Shanghai, China) according to the manufacturer's protocol.

The scratch and cell invasion assays were carried out in accordance with our previous reports 11. This experiment was performed in triplicate.

### Metastasis assays *in vivo*

The OS cells (Including 2.0 × 10^6^ shRNA-RNF187-MG-63 and HOS-RNF187 cDNA cells and their control cells were suspended in 100 μL serum-free RPMI 1640 medium with Matrigel™ (1:1) (BD Biosciences, San Jose, CA, USA), and then injected into the flank of nude mice (s.c.). The implanted tumor volume and total number of lung metastases were evaluated according to our and other previous reports 11, 15.

### Tissue microarrays and immunohistochemistry

Tissue microarrays (TMA) and immunohistochemistry (IHC) and were constructed and performed as our pervious report 11. Polyclonal rabbit anti-human RNF187 (1:200; Novus Biologicals, Cambridge, UK) was used to detect the RNF187 protein. RNF187 expression was evaluated according to the intensity and ratio of positive tumor cells as the previously described 6, 13, 16. The two levels of RNF187 intensity of were classified according to the mean area of positive staining, and the cutoff value was 50% of tumor section, the ≥50 percent was positive, and negative was < 50 percent.

### Statistical analysis

The SPSS 21.0 software (SPSS) was employed to analyze the data. Values were showed as the mean ± standard deviation. The Fisher's exact probability, χ^2^ test, and Student's t test were used for comparison between groups. Overall survival and recurrence free survival (RFS) were defined as described previously 20. Prognostic significance was analysed by Kaplan-Meier survival analysis and log-rank tests. The *P* <0.05 was considered statistically significant.

## Results

### RNF187 expression is elevated in OS tissues

**Figure 1:**
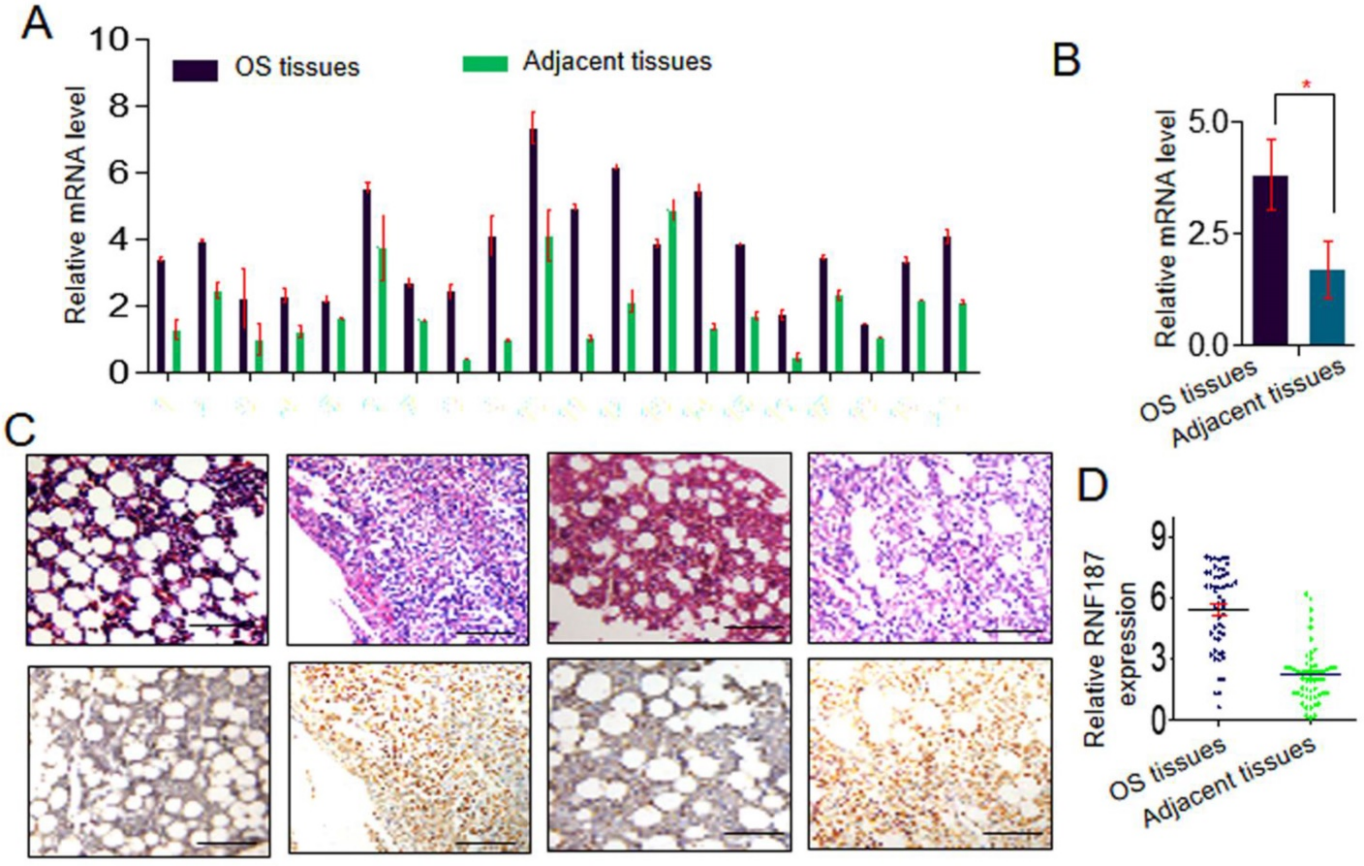
** The RNF187 expression in os. A and B.** qRT- PCR analysis of the RNF187 expression in OS tissues and matched nontumorous tissues, Data are showed as the mean ± SD, n=3. **C and D.** Representative H&E and RNF187 expression in OS tissues and matched nontumorous tissues (Bar=200μm).

Expression of RNF187 was examined by qRT-PCR in OS and matched nontumorous tissues. Low expression of RNF187 was detected in matched nontumorous compared with OS tissues. As shown in **Fig. 1A** and **1B**, the relative RNF187 expression was 3.83 ± 0.79 and exhibited considerable variation in OS samples (range 1.28 - 6.27), while mean expression level was only 1.70 ± 0.63 in matched nontumorous tissues (range 0.59 - 2.64). The difference in RNF187 expression between OS and matched nontumorous tissues was statistically significant (*p* < 0.01). IHC also showed a high level of RNF187 in OS samples compared with matched nontumorous tissues (**Fig. 1C and 1D**).

### RNF187 promotes metastasis and invasion of OS *in vitro*

**Figure 2:**
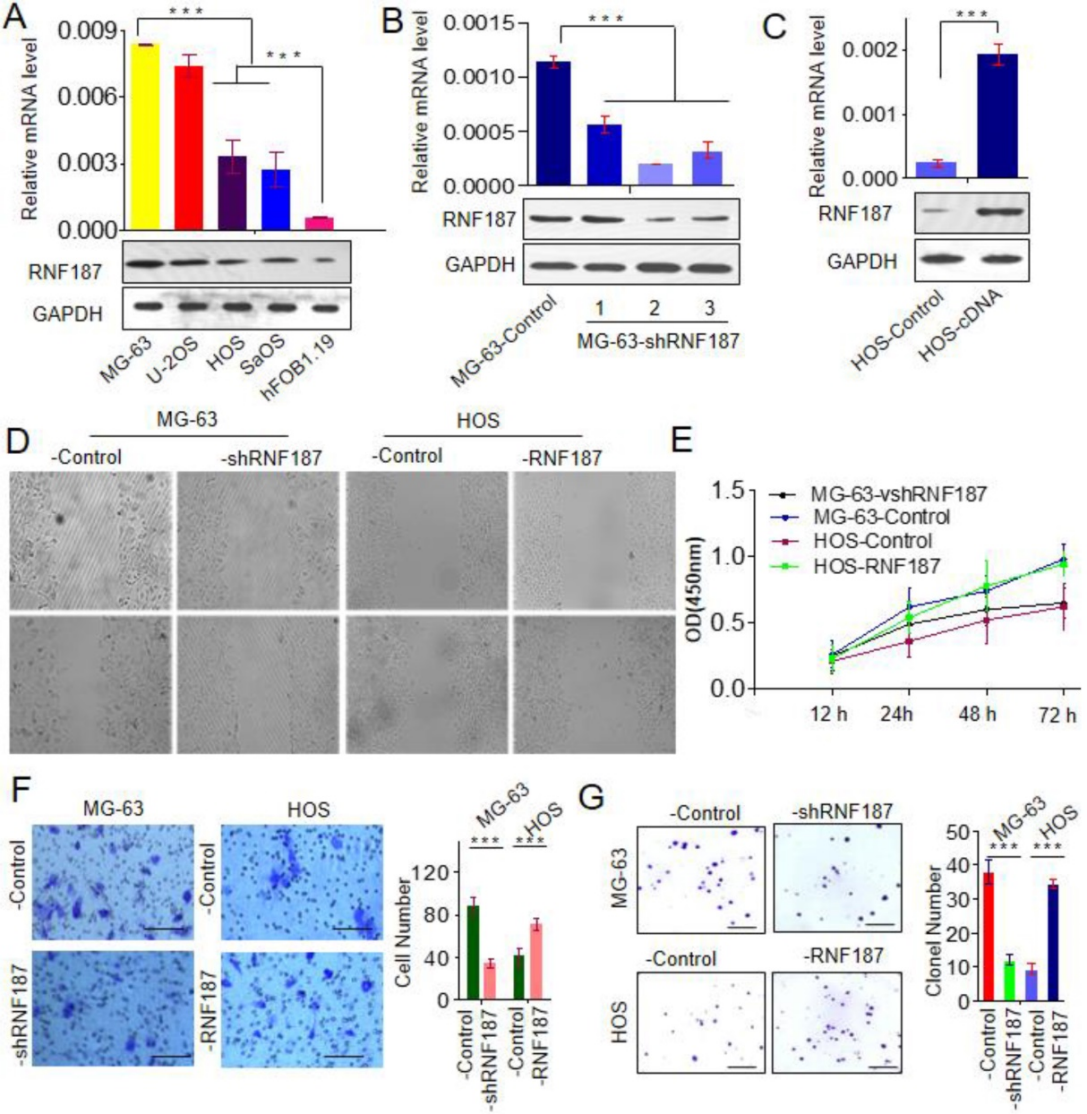
** High level of RNF187 promote OS progression. A.** Western blot and qRT- PCR analysis of the RNF187 expression in OS and hFOB1.19 cell lines; **B.** RNF187 expression was effectively interfered in MG-63 cells by specific RNF187-shRNA vectors; **C.** RNF187 expression was up-regulated in HOS cells by transfecting the RNF187 cDNA vectors; **D.** wound‐healing assays were used to evaluate the migration of OS cells with different RNF187 expression;** E.** Cell proliferation in OS cells with enhanced or reduced RNF187 expression was assessed by a CCK-8 assay.** F.** Transwell assays were used to measure the effects of RNF187 up- and down‐regulation on the invasion of OS cells; **G.** Changes in Colony formation activity of OS cells with RNF187 up- and down‐regulation.

To explore the biological role of RNF187 in OS, we firstly evaluated the RNF187 expression in hFOB1.19, Saos, HOS, U-2OS and MG-63 cell lines, and found that RNF187 expression in OS cells was higher than that in hFOB1.19 cells at the level of mRNA and protein (*p* < 0.05). Then MG-63 cells were transfected with pGPU6-GFP-vshRNA-RNF187s. Of three vshRNA-RNF187s tested, #2 was found to be the most efficient downregulation of RNF187 by qRT-PCR and western blot assays. The pGMLV-RFP-cDNA-RNF187 vectors were transfected to HOS cells, and the RNF187 was obviously up-regulated in HOS cells (**Figs. 2 A, B and C**) and selected for following experiments. The OS cells proliferation were inhibited by the interference of RNF187, while increased by RNF187 cDNA transfection (*p* > 0.05, **Fig. 2D**). The scratch assay showed that an distinctly postponement in the wound closure rate of MG-63-shRNA-RNF187 and HOS-RNF187 cells was found at 48 h, compared with their control cells (**Fig. 2E**). The transwells assay showed that down-regulated RNF187 expression was associated by weaken invasiveness of MG-63 a cells, while was enhanced by RNF187-cDNA transfection (**Fig. 2F and 2G**). Moreover, the cells with high level of RNF187 showed enhanced ability of clone formation (**Fig. 2H and 2I**). These results indicated that overexpression of RNF187 was along with increased metastatic and proliferative potential of OS cells.

### Elevated RNF187 induces the drugs resistance of OS cells, increased the activation of ERK1/2 and BCL-2 expression

**Figure 3:**
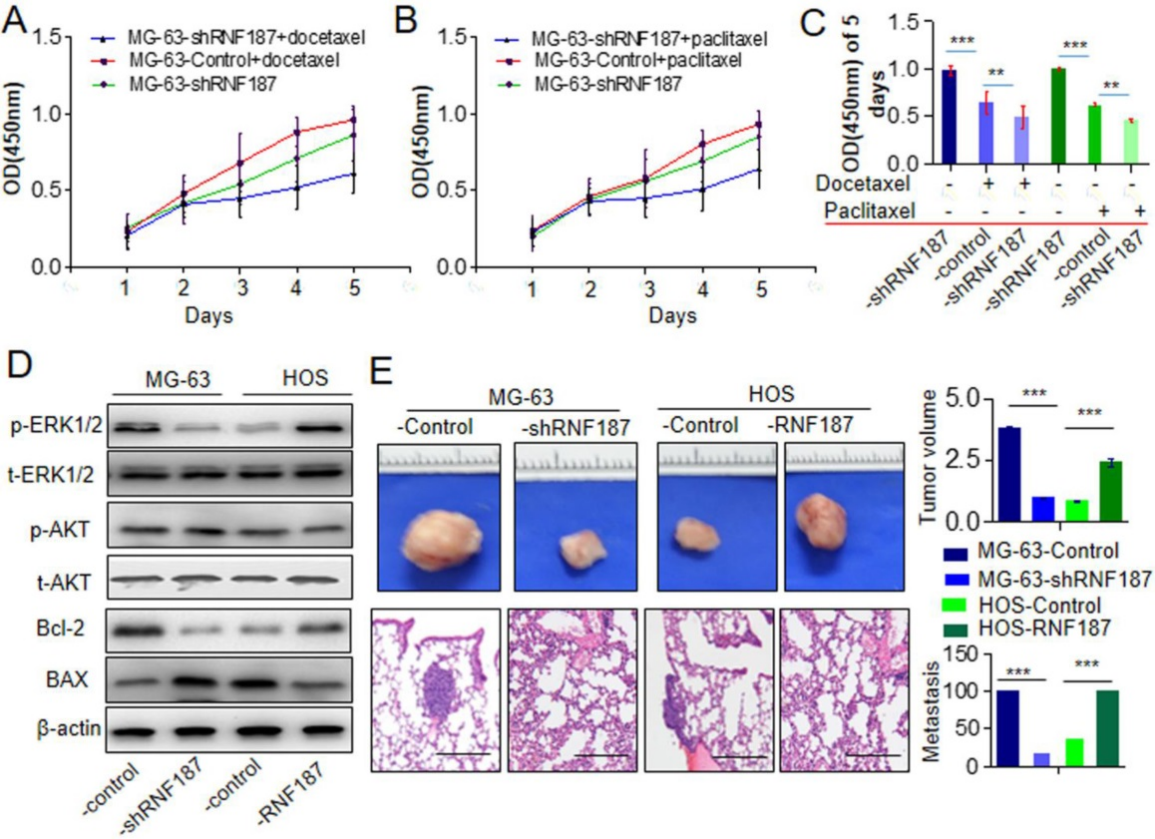
** High level of RNF187 induce drug resistance and metastasis *in vivo*. A and B**. The cell viability was investigated at the cells treated by paclitaxel and docetaxel. **P<0.01.** C.** The cell viability was investigated at the cells treated by paclitaxel and docetaxel in 5^th^ day. **P<0.01.** D.** Western blot was used to detect the ERK1/2 and AKT signaling- and apoptosis related molecules.** E.** The volume of the tumors derived from OS cell lines with different RNF187 expression was calculated *in vivo* for 6 weeks; Serial sections from mouse lung showed the metastasis ability of cancer cells expressing different RNF187 (Scale bar: 50 μm).

The chemoresistance to anti-OS therapy is one of the major obstacles in the treatment of OS, including paclitaxel and docetaxel which were routinely used in the therapy of OS 3, 17, 18. Here, we further explored the roles of RNF187 on the effect of paclitaxel and docetaxel in OS cells. We found that proliferation decreased significantly upon treatment with paclitaxel and docetaxel in OS cells with low level of RNF187 compared with the OS cells with high level of RNF187(**Figure 3A and B**). Additionally, we revealed that high level of RNF187 was associated with the activation of ERK1/2 signaling, and elevated expression of BCL-2(**Figure 3C**). *In vivo* analysis showed that the tumor volumes of in the MG-63-shRNA-RNF187 or HOS group were smaller compared to their controls (*p*<0.05, **Figure 3 D**), and the incidences of lung metastasis were 40% and 20% in the MG-63-shRNA-RNF187 and HOS groups compared to 100% in the control groups, respectively **(Figure 3E).**

### Expression of RNF187 was positively associated with malignant phenotypes of OS

**Figure 4:**
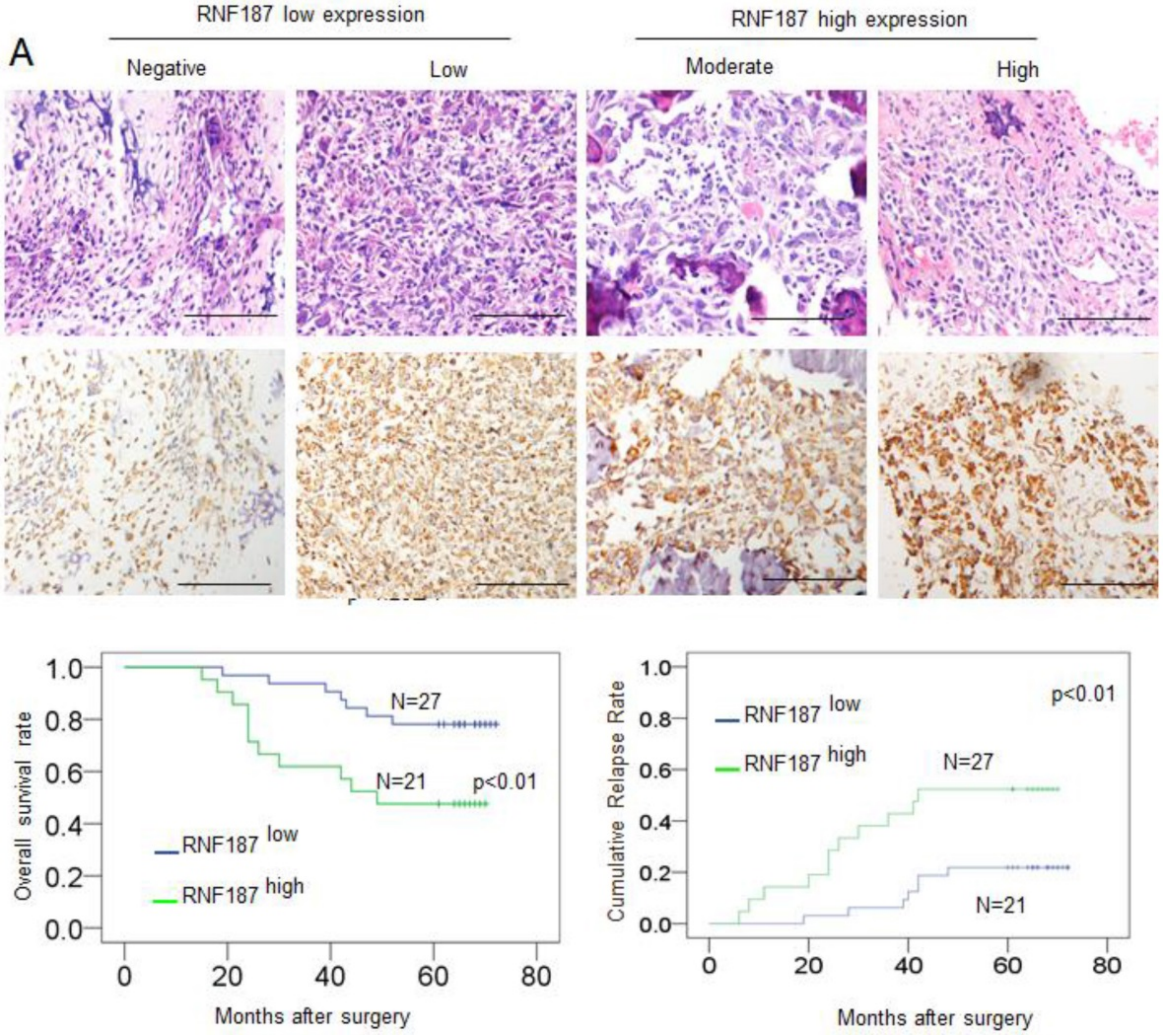
** The RNF187 overexpression in** OS** prognosis. A.** The representative pictures of RNF187 expression in OS tissues; **B.** Survival analysis of 51 OS patients with respect to RNF187 expression.

Positive RNF187 staining was located in the cytoplasm of tumor cells and showed substantial heterogeneity in the different tumor specimens **(Figure 4A)**. A proportion of 47.05% (24/51) was RNF187^high^ in the total number of patients. Patients with high RNF187 expression exhibit aggressive phenotype. As shown in **Table 1**, RNF187^high^ was significantly correlated with high Enneking stage (*p* = 0.001), a poor response to chemotherapy (*p* = 0.004) and metastasis (*p* = 0.001) compared to the patients with low expression of RNF187. However, additional clinical features, containing age, sex, and site of primary tumor, were not significantly relevant to the RNF187 expression.

### Overexpression of RNF187 was associated with poor prognosis of OS patients

Up to the final follow-up, the 5-year overall survival and relapse-free survival (RFS) in the whole population were 69.44%, and 62.35%, respectively. The 2- and 5-year overall survival in the RNF187^low^ group was apparently higher than that in the RNF187^high^ group (**Fig. 4B**). The 2- and 5-year RFS in the RNF187^low^ group were apparently higher than those in the RNF187^high^ group (**Fig. 4C**), indicating that RNF187 expression predicts an unfavorable prognosis for patients with osteosarcoma. Univariate analysis showed that overexpression of RNF187, high Enneking stage, poor response to chemotherapy, and lymphatic metastasis were predictors of overall survival and RFS. Additional features containing age and sex had no prognostic significance for overall survival or RFS (**Table 2**).

## Discussion

OS has a strong tendency to metastasize, and metastasis is the key cause of therapy disappointment and death for OS patients 19. In this study, we showed that RNF187 overexpressed in OS compared to matched nontumorous tissues. By RNA interference and cDNA transfection, we presented that OS cells with high levels of RNF187 appeared the high invasive and metastatic potential both *in vitro* and *in vivo*. Furthermore, we also confirm that OS cells expressing high levels of RNF187 showed drugs resistance to chemotherapeutic agents. Clinically, we found that the elevated expression of RNF187 correlates with poor survival and with disease recurrence in TMA including 51 OS cases. The above results indicated RNF187 functioned as a promoter for the proliferation and invasion of RNF187-overexpressing OS cells.

Now, it is acknowledged that ubiquitination plays an important role in posttranslational protein modification, regulating a host of crucial cellular processes, such as cell cycle, apoptosis, and DNA repair 20, 21. Thus, it is easy to understand that dysfunction of ubiquitin has been identified to be related to the tumorigenesis and progression of various tumors 20, including lung cancer 22-24, colorectal cancer 25, hepatocellular carcinoma 26, 27 and OS. Our results indicated that high level of RNF187 promote OS progression, which was concluded the following facts. Firstly, the expression of RNF187 is higher in OS tissues and cells than that in matched nontumorous tissues and the fetal osteoblastic cell, and low level of RNF187 decreased accordingly the capacity for tumor metastasis and invasion both *in vitro* and *in vivo*. Secondly, we show that high expression of RNF187 is associated with drugs resistance in OS cells. Lastly, we demonstrate that RNF187 overexpression occurs more frequently in various OS with poor prognosis-associated clinical variables. Importantly, we also show that elevated expression of RNF187 is correlated with poor survival and early disease recurrence in a cohort of OS patients. Thus, we consider that RNF187 overexpression promotes OS progression.

In summary, our findings identify RNF187 as a predictor of overall survival and recurrence in patients with OS, and RNF RNF187 may be a potential therapeutic target for OS patients.

## Supplementary Material

Supplementary table S1.

## Figures and Tables

**Table 1:** Correlation between RNF187 expression and OS clinicopthological parameters

		Result of immunostaining (No. of patients)		
Parameter		RNF187-negative (n =27/51)	RNF187-positive (n=24/51)	*p*
**Age (yrs)**				
	<19	17	14	
	≥19	10	10	0.937
**Gender**				
	Male	18	16	
	Female	9	8	0.052
**Site of primary tumor ^†^**				
	Femur	19	15	
	Tibia	4	4	
	Humerus	3	3	
	Pelvis	0	1	
	Other	1	1	0.103
**Histologic differentiation ^††^**				
	Osteoblastic	14	22	
	Chondroblastic	2	2	
	Fibroblastic	7	0	
	Telangiectatic	3	0	
	Other	1	0	0.001
**Enneking stage ^‡^**				
	I	3	0	
	IIA	8	2	
	IIB	15	17	
	III	1	5	0.001ª
**Response to chemotherapy** ^*^				
	Good	20	8	
	Poor	5	16	
	NA	2	0	0.004
**Metastasis**				
	Negative	18	6	
	Positive	9	18	0.001

**Note:** ^†^ Femur *vs.* Tibia/ Humerus/Pelvis/Other; ^††^ Osteoblastic *vs.* Chondroblastic/ Fibroblastic/ Telangiectatic/Other; ^‡^ I/IIA* vs.* IIB/III; ^*^ good* vs.* poor/NA; ª Fisher's exact probability.

**Table 2:** Univariate analysis of factors associated with OS survival and recurrence

Variables	Overall survival		RFS	
	Hazard ratio (95% CI)	*p* value	Hazard ratio (95% CI)	*p* value
Sex (Male* vs.* Female)	1.863 (0.590-4.670)	0.317	1.458(0.519-4.096)	0.413
Age (years) (<19 *vs.* ≥19)	1.119 (0.983-1.037)	0.502	1.012 (0.986-1.040)	0.311
Enneking stage (I/IIA *vs*. IIB/III)	3.476 (1.157-9.312)	0.017	4.225 (1.328-9.924)	0.029
Site of primary tumor (Femur* vs*. other)	1.175(0.378-8.202)	0.974	1.377 (0.417-2.784)	0.762
Response to chemotherapy (good* vs*.poor/NA)	4.326 (1.125-8.153)	0.013	3.712(1.631-7.531)	0.002
Metastasis (negative *vs.* positive)	12.002 (1.463-83.627)	0.001	37.148 (3.652-298.156)	0.001
RNF187^low^ *vs.* RNF187^high^	3.172(1.085-7.083)	0.023	3.719 (1.316-11.710)	0.009

**Abbreviations and Note:** OS, Osteosarcoma; RFS, relapse-free survival; 95%CI, 95% confidence interval; HR, Hazard ratio; Cox proportional hazards regression model.
