# Supplementary material for: High level of RNF187 contributes to the progression and drug resistance of osteosarcoma
Source: J Cancer. 2020 Jan 1;11(6):1351–8. doi: 10.7150/jca.33488 (PMC6995399; doi:10.7150/jca.33488)
Supplement: Supplementary file 1 — Supplementary table S1. [file jcav11p1351s1.pdf]

Table S1. The primary antibodies for western blot and IHC

Antibody	Concentration for WB	Concentration for IHC	Concentration for IF	Specificity	Company
RNF187	1:1000	1:100	1:100	Polyclonal rabbit	Novus Biologicals
AKT	1:1000	/	/ /	Rabbit polyclonal	Cell Signaling Technology
p-AKT^{s473}	1:1000	/	/ /	Rabbit polyclonal	Cell Signaling Technology
ERK	1:1000	/	/ /	Rabbit polyclonal	Cell Signaling Technology
p-ERK	1:1000	/	/ /	Rabbit polyclonal	Cell Signaling Technology
Bax	1:1000	/	/ /	Rabbit polyclonal	Cell Signaling Technology
Bcl-2	1:1000	/	/ /	Rabbit polyclonal	Cell Signaling Technology

Abbreviations: WB, western blot; IHC, immunohistochemistry; CO-IP, immunoprecipitation; IF, immunofluorescence
